# Safety and effectiveness of recombinant human growth hormone replacement in postoperative craniopharyngioma children

**DOI:** 10.1186/1687-9856-2013-S1-P63

**Published:** 2013-10-03

**Authors:** Zhang-qian Zheng, Di-jing Zhi, Shui-xian Shen, Fei-hong Luo, Zhu-hui Zhao, Zhong Lu, Rong Ye, Ruo-qian Cheng, Xiao-jing Li

**Affiliations:** 1 Department of Endocrinology and Inherited metabolic disease, Children’s Hospital of Fudan University, Shanghai, 201102, China

## Aims

To observe the safety and effectiveness of recombinant human growth hormone (rhGH) replacement therapy (GHRT) in postoperative craniopharyngioma (CP) children.

## Subjects and methods

We reviewed the records for all patients undergoing GHRT at our hospital over the study period. Patients were included if they had received CP resection, GHRT for at least 12 months, and records of serial magnetic resonance imaging data and data for treatment, pituitary hormone profiles and growth chart were available. GH-naïve control patients were selected from our hospital database of patients carried out the same surgery. Patients were matched for date of surgery, age, site of primary diagnosis and sex.

## Results

18 patients were recruited with growth hormone deficiency. In treatment group, all patients all gained acceleration in growth velocity and elevated growth factors level. There were no recurrent tumors found in both groups.

## Conclusions

Our study demonstrates no increased risk for recurrent in patients receiving GHRT, thus supporting a high safety profile of GHRT in postoperative craniopharyngioma children. Additionally, GHRT can provide a significantly change in growth velocity compared with control group.

